# Book Review: Handbook of Positive Youth Development: Advancing Research, Policy, and Practice in Global Contexts

**DOI:** 10.3389/fpsyg.2021.749385

**Published:** 2021-09-03

**Authors:** Denisse Manrique-Millones

**Affiliations:** Grupo de Investigación en Comunicación y Salud, Instituto de Investigación Científica, Universidad de Lima, Lima, Peru

**Keywords:** Positive Youth Development, promotion, public health, prevention, multidisciplinary

The *Handbook of Positive Youth Development: Advancing Research, Policy, and Practice in Global Contexts* edited by Profs. Radosveta Dimitrova and Nora Wiium combine theory, empirical research, and practical considerations for relevant policy and practice. The volume includes 37 distinctive chapters covering a multitude of theoretical and methodological approaches with youth and young adults from diverse cultures, ethnics, and sociodemographic backgrounds. With unique samples and global approaches to research, policy, and practice, this volume presents the most inclusive collection of Positive Youth Development (PYD) among youth and emerging adults to date.

The introductory chapter provides an overview of the main models of PYD, namely, the developmental assets (Scales et al., 2017), the 5Cs and 6Cs models of PYD (Geldhof et al., 2015), and the newly developed 7Cs model of PYD among young people in a variety of cultural contexts (Abdul Kadir et al., 2021; Dimitrova et al., 2021; Manrique-Millones et al., 2021). A rationale is then provided, with information about sections and contributions within multidisciplinary disciplines of psychology, public health, developmental and environmental science, sociology, family and youth studies, and prevention. The major objectives of the volume are to (1) advance the theoretical and empirical knowledge base on PYD among young people; (2) expand understanding on methodological issues and measurements on PYD globally; (3) integrate PYD scholarship with relevant research, policy, and practice.

This volume begins with a *foreword* by Daniel Shek (The Hong Kong Polytechnic University), one of the leading scholars in PYD research and intervention, especially in Asia. In his brief overview, Shek summarizes several unique features and relevant conceptual and methodological contributions. Next, all 37 chapters are organized along with two major categories with empirical contributions, including a substantial number of youth and emerging adults (*N* = 22,083) in major geographical continents, many of which are neglected in the research literature on adolescence. Part I, *Positive Youth Development in Global Contexts;* Part II, *Positive Youth Development Applications and Interventions* were structured to represent first, the most neglected cultural contexts and populations around the globe. The final chapter by John Geldhof, Svea Olsen, and Asia Thogmartin (Oregon State University, USA), leading PYD scholars in the U.S. and globally, represents an overview of the contributions to the PYD field, with a relevant outlook for research, policy, and practice. This volume represents a valuable scholarly resource for students, researchers, lecturers, and practitioners, interested to promote and advance PYD scholarship globally and specifically in underrepresented contexts.

The editors recommend this exciting volume to students, researchers, specialists, and stakeholders, such as policymakers who are committed to improving the lives of young people globally. This *handbook* may be useful for social scientists, psychological, mental and public health professionals, researchers, practitioners, and youth leaders interested in promoting PYD from a variety of disciplines (e.g., positive psychology, well-being studies, developmental psychology, child and family studies, cross-cultural psychology, education, prevention, intervention, intercultural relations, social psychology, anthropology, sociology, methodology, counseling, community psychology, emerging adulthood and applied developmental science).

## Author Contributions

The author confirms being the sole contributor of this work and has approved it for publication.

## Conflict of Interest

The author declares that the research was conducted in the absence of any commercial or financial relationships that could be construed as a potential conflict of interest.

## Publisher's Note

All claims expressed in this article are solely those of the authors and do not necessarily represent those of their affiliated organizations, or those of the publisher, the editors and the reviewers. Any product that may be evaluated in this article, or claim that may be made by its manufacturer, is not guaranteed or endorsed by the publisher.
